# Novel Z-DNA binding domains in giant viruses

**DOI:** 10.1016/j.jbc.2024.107504

**Published:** 2024-06-27

**Authors:** Miguel F. Romero, Jeffrey B. Krall, Parker J. Nichols, Jillian Vantreeck, Morkos A. Henen, Emmanuel Dejardin, Frederik Schulz, Quentin Vicens, Beat Vögeli, Mamadou Amadou Diallo

**Affiliations:** 1 DOE Joint Genome Institute, Lawrence Berkeley National Laboratory, Berkeley, California, USA; 2 Department of Biochemistry and Molecular Genetics, University of Colorado at Denver, Aurora, Colorado, USA; 3 GIGA I3 - Molecular Immunology and Signal Transduction, University of Liège, Liège, Belgium; 4 Department of Biology and Biochemistry, Center for Nuclear Receptors and Cell Signaling, University of Houston, Houston, Texas, USA

**Keywords:** innate immunity, Zα domain, B-Z conversion, B-to-Z conversion, ADAR1, ZBP1

## Abstract

Z-nucleic acid structures play vital roles in cellular processes and have implications in innate immunity due to their recognition by Zα domains containing proteins (Z-DNA/Z-RNA binding proteins, ZBPs). Although Zα domains have been identified in six proteins, including viral E3L, ORF112, and I73R, as well as, cellular ADAR1, ZBP1, and PKZ, their prevalence across living organisms remains largely unexplored. In this study, we introduce a computational approach to predict Zα domains, leading to the revelation of previously unidentified Zα domain-containing proteins in eukaryotic organisms, including non-metazoan species. Our findings encompass the discovery of new ZBPs in previously unexplored giant viruses, members of the *Nucleocytoviricota* phylum. Through experimental validation, we confirm the Zα functionality of select proteins, establishing their capability to induce the B-to-Z conversion. Additionally, we identify Zα-like domains within bacterial proteins. While these domains share certain features with Zα domains, they lack the ability to bind to Z-nucleic acids or facilitate the B-to-Z DNA conversion. Our findings significantly expand the ZBP family across a wide spectrum of organisms and raise intriguing questions about the evolutionary origins of Zα-containing proteins. Moreover, our study offers fresh perspectives on the functional significance of Zα domains in virus sensing and innate immunity and opens avenues for exploring hitherto undiscovered functions of ZBPs.

Nucleic acids adopt a variety of structures other than right-handed double helices. Both DNA and RNA can form triplexes, I-motifs, G-quadruplexes, and left-handed helices referred to as “Z-nucleic acids” (Z-DNA and Z-RNA) (1, 2, 3). These alternative structures are critical components of cellular functions, influencing a myriad of biological processes (4, 5, 6). In particular, it is now well established that the innate immune system exploits Z-nucleic acids as pathogen-associated molecular patterns and damage-associated molecular patterns (7, 8).

Z-DNA/Z-RNA biology owes its now-recognized relevance to several proteins containing Zα domains, which are responsible for recognizing Z-nucleic acids (9). Zα domains are found in metazoan proteins involved in immunity and cancer, as well as in viral proteins. The first crystal structure revealed that the Zα domain of ADAR1 (adenosine deaminase acting on RNA 1) adopts a winged-helix-turn-helix (wHTH) fold. This structure is characterized by a compact α/β architecture encompassing a three-helix bundle (α1 to α3), juxtaposed to a twisted antiparallel β-sheet (β1 to β3) (10). Specific amino acids are important for the specific recognition of Z-nucleic acids, such as N173 and Y177 within helix α3, and P192 and P193; W195 contributes to both protein stability and nucleic acid binding (10). This earlier work demonstrated that the commonly found wHTH domain across proteins only binds to Z-nucleic acids when these amino acids are present.

The only other proteins known to contain Zα domains are ZBP1 in mammals, amphibia, and reptilia (11), PKZ in salmoniform and cypriniform fish (12), E3L in poxviruses (13), ORF112 in cyprinid herpesviruses (CyHV-1-to-3) (14) and I73R in the asfarviridae African swine fever virus (ASFV) (15). Our current understanding of the biology of these proteins points to antagonistic relationships in innate immunity and a potential regulatory role in gene expression (16, 17). Briefly, the recognition of Z-nucleic acids by ADAR1 and ZBP1 is essential for the immune system's ability to detect and respond to viral threats effectively (18). Conversely, viruses have evolved Zα-containing proteins like E3L to subvert and antagonize host immune defenses, thereby enhancing their own virulence and survival within the host organism (19, 20). However, Zα domains have functional roles that need to be nuanced, as they cannot always be swapped, especially across different viruses (20, 21). This level of complexity implies an evolutionary balance between a virus and its host, where the unique characteristics of Zα domains are finely tuned to suit the particular dynamics of each virus-host relationship.

Because Z-nucleic acid biology is now viewed as a determinant for cell fate during infection and auto-immune diseases, one could ask the question of whether more proteins with Zα domains could be discovered, and in what species. Recent computational efforts based on structure similarity search have unveiled the existence of 14 potential Zα domains (22). Additionally, two other potential ZBPs were identified, namely RBP7910 in *Trypanosoma brucei* (23), and DprA in *Riemerella anatipestifer* (24). However, these studies did not address the absence of the complete set of required amino acids for binding Z-nucleic acids, and no experimental validations of these putative Zα domains were carried out.

Here, we introduce a computational approach for predicting Zα domains, which combines primary sequence analysis, three-dimensional modeling, and manual examination of the resulting hits through structural analysis. This method led to the discovery of 8 putative Zα domain-containing proteins in eukaryotic organisms, and 68 in giant viruses diverse and mainly unexplored giant viruses in the viral phylum *Nucleocytoviricota*. We also report Zα-like domains in bacterial proteins, which deviate somewhat from their eukaryotic and viral counterparts in primary amino acid sequence. Finally, we have experimentally validated our predictions by assessing the Z-DNA binding capacity and ability to shift the B-Z equilibrium for two Zα and two Zα-like candidates. This work offers fresh insights into the functional role of Zα domains in virus sensing and innate immunity. Furthermore, it may shed light on previously undiscovered functions of ZBPs, expanding our understanding of their roles in biological processes.

## Results and discussion

### A combinatory method to predict Zα-domain and Zα-like candidates

**Figure 1:**
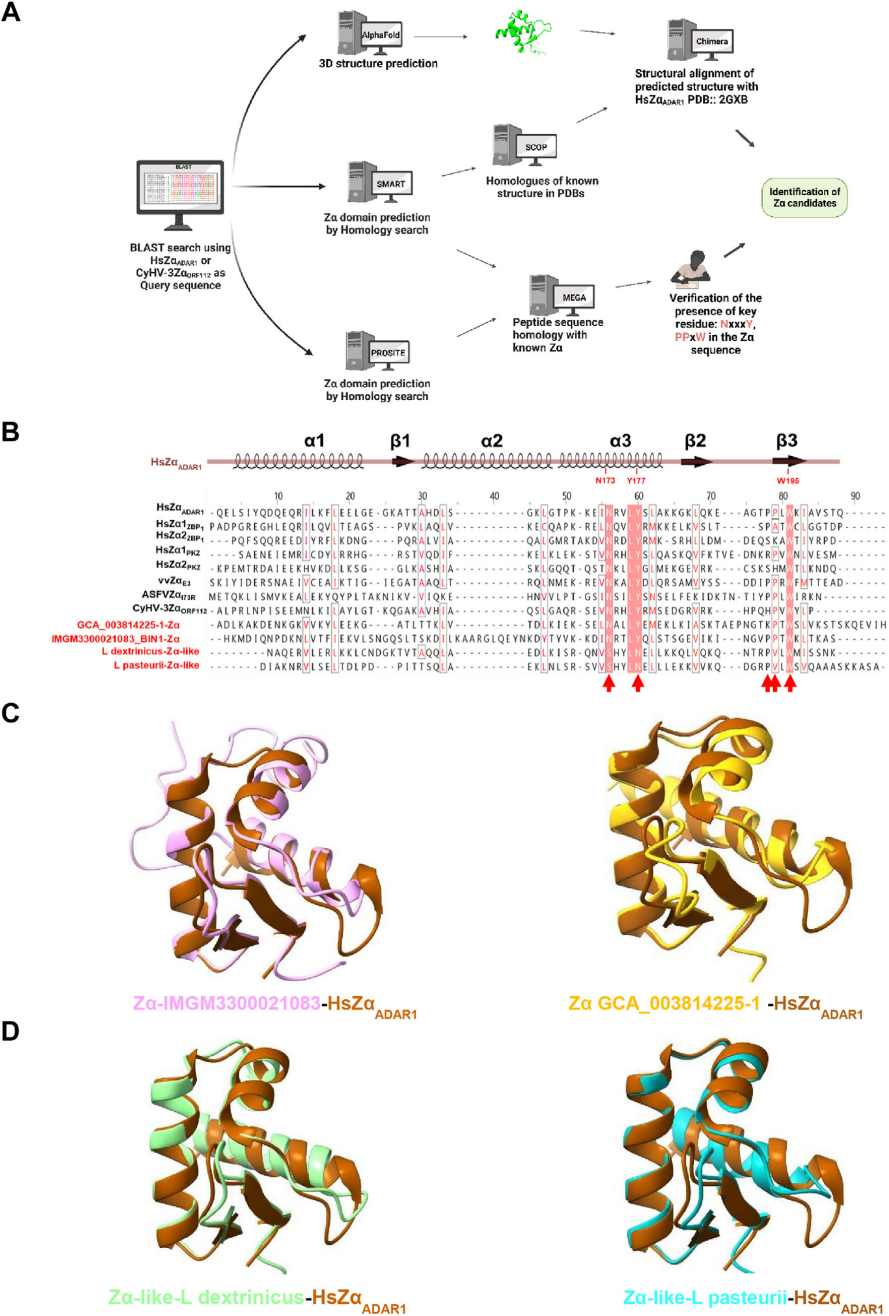
**Prediction of new Zα candidates**. *A*, the summary of the workflow for identifying Zα candidates. *B*, alignment of two predicted Zα and two Zα-like with known Zα domains from human and mouse ADAR1 (HsZα_ADAR1_), human ZBP1 (HsZα_ZBP1_), and viral from vaccinia virus and cyprinid-herpesvirus-3 (vvZα_E3_ ASFV Zα_I73R_ CyHV-3Zα_112_). The consensus structure Zα domain is shown at the *top*. The key DNA-interacting residues in ADAR1 (corresponding to Asn174, Tyr177, P192, P193, and Trp195 in HsZα_ADAR1_) are pointed with *red arrows*. Hydrophobic residues in the hydrophobic core are highlighted in *red front*. *C*, structural alignment of AlphaFold predicted structure of Zα candidates with HsZαADAR1 (*brown*, PDB ID 2GXB). *D*, structural alignment of AlphaFold predicted structure of Zα-like candidate with HsZα_ADAR1_ (*brown*, PDB ID 2GXB).

Initially, we employed BLAST search tools in conjunction with primary sequence analysis by domain prediction software SMART and PROSITE to identify the Zα-domain candidates. Subsequently, 3D structure analysis and prediction tools were used to refine our predictions. Leveraging our existing knowledge of the essential attributes of the Zα-domain, we further refined the results derived from our *in silico* analysis (Fig. 1*A*). We used multiple sequence alignments to compare the candidate sequences with some representative Zα domains in all 6 known ZBPs. We checked the presence of residues crucial for the functionality of the Zα-domain by primary sequence alignment. Among these, the NxxxY motif within α3 is essential for Z-DNA binding, while the pPxW motif in the β-wing plays a significant role in both Z-DNA (10) binding and A-to-Z conversion (25). In the crystal structure of the HsZα_ADAR1_, tryptophan 195 has been demonstrated to be vital not only for protein stability but also for DNA binding. The double mutation involving asparagine 173 and tyrosine 177 within the α3 helix completely abolishes Zα′s capacity to bind Z-DNA, rendering it functionally inoperative for both mammalian and viral ZBPs. Additionally, proline 192 and proline 193 contribute significantly through van der Waals interactions with DNA (10). Despite the limited overall sequence similarity typical to Zα domains, the aliphatic residues (marked in red) from the three helices, along with Tryptophan 195 in the β-wing, exhibit strong conservation in all candidates. These residues are well-established for their role in supporting the hydrophobic core of the winged helix-turn-helix structure (Figs. 1*B*, and S1) (10). In line with this, the predicted 3D structures, generated by AlphaFold (26), reveal that all candidates adopt the characteristic winged-HTH conformation. The 3D fold comparisons underscore a remarkable degree of structural similarity between these candidates and the crystallized ADAR1 Zα domain, PDB ID 2GXB (27) (Fig. 1, *C* and *D*). Despite these similarities, some candidates found in bacteria lack conserved residues in the corresponding positions N173 and Y177 in HsZα_ADAR1_ displaying instead a serine and an asparagine respectively. As such, we classify them as Zα-like candidates (Figs. 1*B* and S1).

### Identification of novel Zα-domain candidates in eukaryotic organisms

All predicted Zα candidates maintain the conserved residues N173 and Y177 in HsZα_ADAR1_ crucial for Z-DNA binding (10). In Anthozoa, a class of marine invertebrates that includes sea anemones, stony corals, and soft corals, we predicted a new Zα containing protein. Interestingly, in some families such as the Poritidae or Actinidea, the novel Zα displays all important residues found in the known Zα. However, some families such as Acroporidae or Caryophylliidae display a Zα domain in which the Y177 is substituted by phenylalanine and W195 is replaced by a tyrosine (Fig. S1). We predicted other potential Zα domain-containing proteins in other eukaryotic organisms out of metazoan and DNA viruses, such as a protein annotated as transcription activator p15 in phytoplankton, and another annotated as acetate-CoA ligase in unicellular microalgae of the Symbiodiniaceae family (Figs. S1 and S3).

### Identification of novel Zα-domain candidates in giant viruses

**Figure 2:**
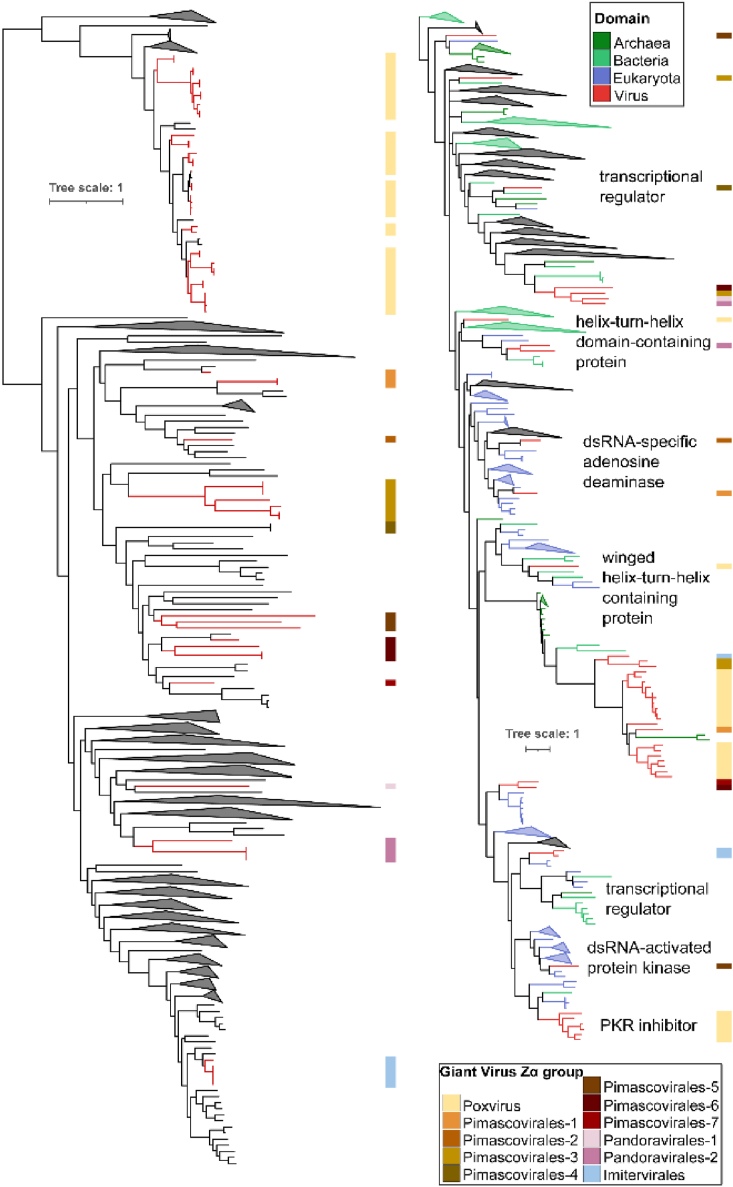
**Phylogenetic tree of proteins that contain Zα and Zα-like domains.** Proteins that were included in the analysis are giant virus ZBPs and homologs identified and extracted from the NCBI nr database.

The most substantial contingent of novel ZBP candidates emerged within giant virus metagenome-assembled genomes (GVMAGs), revealing the presence of potential ZBPs in members of the orders pimascovirales, pandoravirales, and imitervirales. These proteins are distinct from previously documented ZBPs in fish-herpesviruses, poxviruses, and African swine fever virus (ASFV) (Fig. 2). These giant viruses were recovered in our previous metagenomic survey of samples from marine and freshwater, soil, and thermal environments (Fig. S2) (28), but also included additional sequences from deep-sea sediments (29) and permafrost (from 49 to 53,000 years old) (30). Our analysis shows that the candidate ZBPs in these giant viruses share similar domain architectures as in previously identified viral-containing proteins (13, 15, 20). The only exception is the viral ZBP candidate in which Zα is associated with tRNA metabolism domains (IMGM3300021083) (Fig. S3).

In our phylogenetic analysis, the clade topology in the ZBPs protein tree does not align with the topology found in the *Nucleocytoviricota* species tree (Fig. 2). This finding suggests that proteins with Zα domains have been independently acquired, likely multiple times, originating from a specific host *via* gene transfer. This possibility, as proposed by Kuś *et al.*, suggests that CyHV-3 ORF112 might have been co-opted independently from the host by the common ancestor of the cyprinid herpesviruses, rather than through horizontal transfer from a poxvirus (31). Such scenarios are further supported by the fact that in our tree, proteins from different giant virus lineages group together and are simultaneously intertwined in monophyletic clades with eukaryotic and archaeal homologs.

Our results imply that giant viruses with the Zα domain likely infect a wide range of organisms ranging from dinoflagellates to marine vertebrates and arthropods as well as early diverging metazoans such as cnidarians (32). The presence of ZBP in diverse *Nucleocytoviricota* lineages raises the question of the role of Zα in the interactions of these viruses with their hosts, and how this fits into our current understanding of the role of ZBPs in animal innate immunity. Furthermore, the manifold presence of ZBPs in divergent giant virus lineages, known to infect protists and algae, sparks curiosity. This observation is particularly intriguing given that virus-protist interactions entail mechanisms distinctly different from the innate immunity found in animals (33). Could the presence of ZBP indicate that these giant viruses infect metazoan hosts? In line with this, the ASFV infects two phylogenetically distant hosts, functioning as an arthropod-borne DNA virus (34), significantly impacting pig farming due to its high virulence (19).

### Experimental validation of Zα and Zα-like candidates

**Figure 3:**
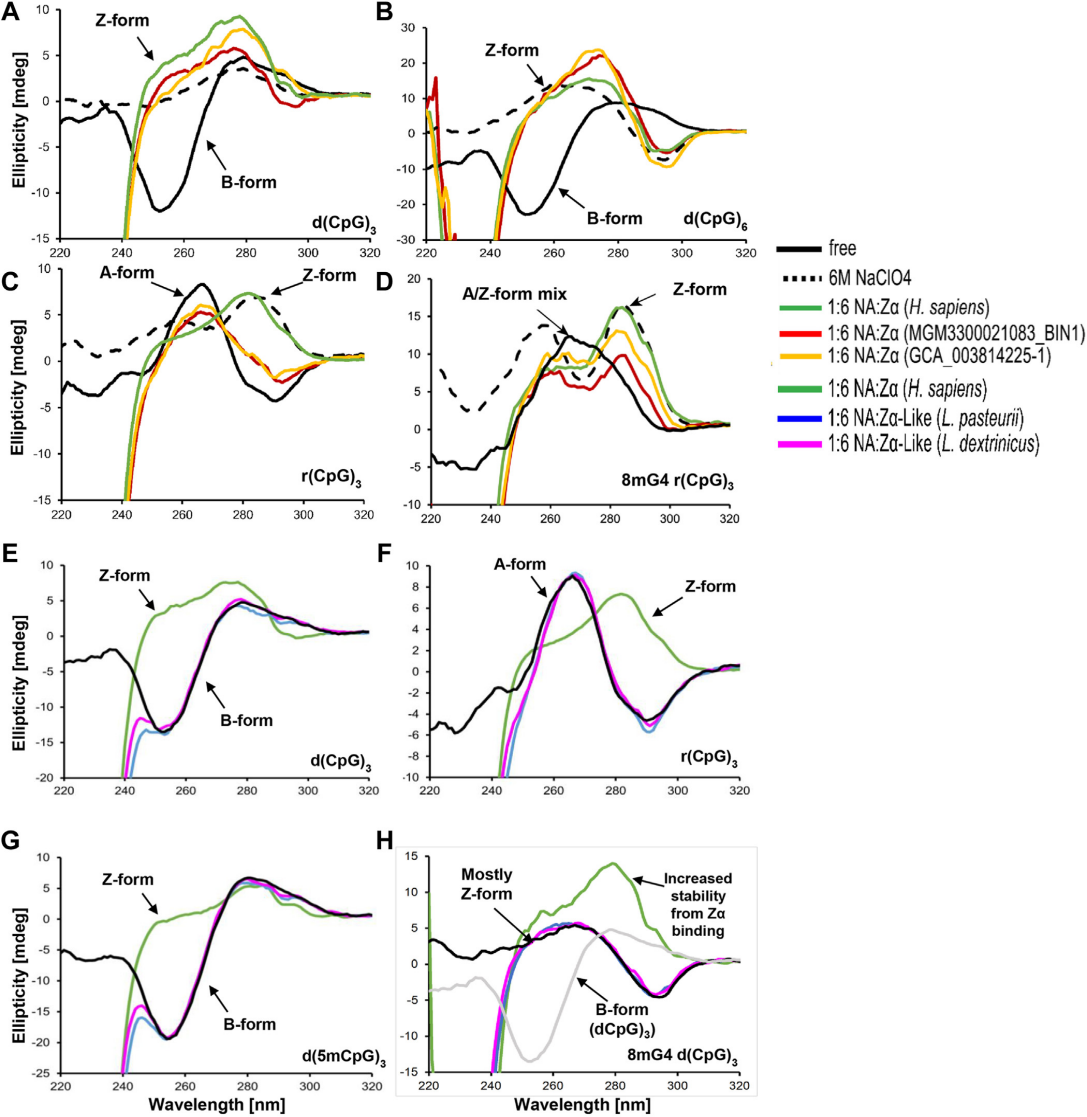
**Experimental validation by circular dichroism**. *A* and *B*, the predicted viral Zα domains induce B-to-Z conversion of DNA. *C*, viral Zα domains do not convert A-RNA to Z-RNA. *D*, viral Zα domains do convert an RNA with prestabilized Z-conformation (A:Z form ∼50%/50%) to full Z-RNA. *E* and *F*, Zα –like domains are not able to induce B(A)-to-Z conversion. *G* and *H*, Zα –like domains do not bind to Z-nucleic acids. For comparison, the profile of a B-form DNA is shown in *grey* in (*H*), demonstrating the presence of a large portion of Z-form in 8mG4 d(CpG)3. The profiles overlaid with profiles from second independent measurements are provided in Fig. S4.

To validate our prediction, we experimentally studied some of these Zα and Zα-like domains by circular dichroism, an established method to probe their ability to induce B(A)-to-Z conversion (35). We found that two predicted Zα domains from two distinct members of the viral order pimascovirales are able to efficiently convert B-DNA duplex of different lengths d(CpG)_3_ and d(CpG)_6_ to Z-form (Fig. 3, *A* and *B*). Interestingly, we found that these two Zα domains are not able to induce A-to-Z conversion of RNA duplex as seen for the positive control HsZα_ADAR1_ or in the high salt concentration condition (Fig. 3*C*). Consequently, our study identifies new cases of Zα capable of converting B-DNA to Z-form but not A-RNA. Indeed, the conversion of A-RNA to Z-RNA is a less favorable and energetically costly process compared with B-DNA to Z-DNA conversion (35). To test the binding capacity to sequence contexts that have a lower B-Z energy barrier or preformed Z-form nucleic acids, we used a singly methylated RNA duplex 8mG4 r(CpG)_3_ which stabilized 50% of the population in Z-form (36, 37) (Fig. 3*D*). In this case, the Zα domains are capable of flipping the RNA to Z-form. This indicates that Zα domains bind to the pre-stabilized Z-RNA since they cannot flip A-RNA to the Z-form.

It is interesting to speculate on the distinction between competent A/B-to-Z flipping Zα domains, Zα domains that can only flip B-DNA, and the nonfunctional Zα-like domains, and their evolutionary/functional relevance. Numerous studies have tried to identify key residues/regions within the Zα domain that are important for binding and flipping nucleic acids to the Z-conformation. Kim *et al.* were able to restore the partial function of ADAR1’s Zβ domain by mutating Ile335 back to a tyrosine as is typical for all other ZBDs (38). Additionally, residues within the β-wing play a large role in not only the stabilization of the Z-conformation but also the rate at which it converts nucleic acids to the Z-conformation (39, 40). Furthermore, distantly related wHTH domains with no ability to convert nucleic acids to the Z-conformation can be converted into good Z-binders and converters through directed mutagenesis (41). It is likely that numerous factors contribute to the ability of any given Zα domain’s ability to bind and convert nucleic acids to the Z-conformation, and the loss of one stabilizing interaction may result in the inability to convert A-RNA while still retaining the ability to convert B-DNA.

As expected, we found that for the Zα-like domain, there was no conversion of B-DNA to Z-DNA or A-RNA to Z-RNA, in contrast to our positive control HsZα_ADAR1_ (Fig. 3, *E* and *F*). To test the binding capacity to sequence contexts that have a lower B-Z energy barrier or preformed Z-form nucleic acids, we used a methylated DNA duplex d(m^5^CpG)_3_ and the singly methylated RNA duplex 8mG4 d(CpG)_3_ respectively (36, 37). The Zα-like domains show no ability to stabilize or shift the B-Z transition equilibrium of the Z-nucleic acid analogs (Fig. 3, *G* and *H*). The Zα-like domain is found in the bacterial protein annotated as sigma 54 (σ^54^)-interacting transcriptional regulator. σ^54^ also encompasses an ATPase AAA region, which overlaps with the Holliday junction DNA helicase RuvB. RuvB is a component of the RuvABC revolvasome, crucial for resolving Holliday junctions (42). Remarkably, both Holliday junctions and Z-DNA are found in bacterial biofilms (43, 44). Studies have shown that RuvA protein or DNABII stabilizes Holliday junctions, inducing supercoiling that reduces the energy needed for B-Z transition and promotes Z-DNA formation in the extracellular DNA biofilm matrix (43). Therefore, it would be interesting to test the B-to-Z conversion of the predicted association between the Zα-like domain and the helicase RuvB in σ^54^. Moreover, knowing that bacteria of the genius of *Lactobacillus* are biofilm-forming bacteria (45), we hypothesize that σ^54^ could be involved in the biofilm matrix formation.

## Experimental procedures

### *In silico* prediction of Zα domain

To identify new Zα domain candidates, we developed a combinatorial approach. Using the Basic Local Alignment Search Tool, blastp algorithm of NCBI (46), we performed taxid-exclusion blast and taxid-targeted blast using the Zα peptides from CyHV-3 and HsADAR1 as the query sequences. The candidates with a minimum of three crucial residues conserved or featuring less drastic substitutions (such as in *A digitifera*-Zα, where phenylalanine replaces the tyrosine in helix-α3 and a tyrosine replaces the tryptophane in the β-wing Fig. S1), were considered as the best hits and were selected. Their primary sequences were subjected to Zα domain prediction using SMART (47) and PROSITE (48, 49) software enabling us to determine the peptide length covered by the Zα domain. Leveraging our knowledge of the essential attributes of the Zα-domain, we further refined the results derived from our *in silico* prediction. We check the presence of certain residues crucial for the functionality of the Zα-domain by primary sequence alignment using MEGA-11 (50) and UGENE (51) tools. The presence or absence of these critical motifs served as the final determinant in our analysis, allowing us to confidently identify Zα candidates. Any candidates lacking tyrosine and asparagine were categorized as Zα-like, given their alignment with other defining characteristics. SMART software also outputs structure primary sequence homologs of known in the protein data bank (PDB) or blast with known SCOPs (structural classification of proteins) of the Zα, for example, **d1qbja** (52). Finally, to strengthen the Zα prediction, a structure prediction using AlphaFold (26, 53) confirms the winged helix-turn-helix fold superposable with the crystallized Zα available with the PDBs using UCSF ChimeraX (54). With the predicted 3D structures, we performed a 3D-BLAST search (55) to confirm that the best hits are entries corresponding to the Zα domain. Figures 1*A* and S3 were created with BioRender.com.

### Sequence source and annotation

Most of the giant viruses used in our analysis were identified in our metagenomic analysis performed in samples from different biotopes (**S2**) (28). We also included other giant viruses identified in other studies such as Pithovirus (29) and Marseillesvirus (30). The sequencing of the full viral genome or the viral genome fragment covering the coding sequence of the ZBP allowed the complete identification of the open reading frame corresponding to the ZBP. For the other organisms, the ZBPs are annotated at the PDB genomic level and (protein and mRNA level for *Micromonas pusilla* (56). Zα domain sequence and the ID numbers of ZBPs are listed in Table S6.

### Phylogenetic analysis

Novel Zα and Zα-like domain sequences were clustered at 40% identity using cd-hit (57) and the resulting cluster representative sequences were aligned using mafft-linsi (version 7.505) (58). We used the hmmbuild (version 3.3.2, hmmer.org) to create a profile hidden Markov model from the representative sequence alignment and then used it as a query for the hmmsearch program (version 3.3.2, hmmer.org) on the NCBI nr database (59). Protein sequences that had an overall e-value <0.005 and contained at least one domain with length ≥ 60 amino acids were considered as significant hits to the model. Then we de-replicated the protein sequences with significant hits with cd-hit at 90% identity and an alignment length covering at least 60% the length of the longer sequence (-aL 0.6). The representative protein sequences were aligned together with Zα containing sequences from the viral phylum Nucleocytoviricota using the mafft FFT-NS-2 method (58). The alignment was trimmed with trimal with the -gt 0.1 option (60). A protein tree was built using IQ-Tree (version 1.6.12) (61) with 1000 ultra fast bootstrap replicates (62) and the JTT+R10 model, which was selected with the ModelFinder feature in IQ-Tree (63). The tree was visualised using iToL (64).

To build the giant virus species tree the nsgtree pipeline was used (https://github.com/NeLLi-team/nsgtree) on representative genome of the viral phylum Nucleocytoviricota (65) and additional GVMAGs that contained proteins with a Zα domain. In brief, 7 giant virus orthologous groups (GVOGs) were identified using hmmsearch (version 3.1/b2, hmmer.org), extracted GVOGs were aligned with mafft (version 7.31) (58), trimmed with trimal (-gt 0.1, v1.4) and concatenated. A species tree was built from the supermatrix alignment using IQ-Tree (version 2.03) (66) with LG+F + I + G4 and visualized in iToL (64).

### Protein expression and purification

The Zα domains from *Homo sapiens* ADAR1, L. pasteurii, *L. dextrinicus*, giant virus (IMGM3300021083 and GCA_003814225–1) were cloned into the pet-28a(+) plasmid (N-terminal 6x His-tag and thrombin cleavage site between His tag and the Zα sequence). The plasmids were transformed and expressed in BL21(DE3) *E. coli* cells. The cell cultures were grown in Luria Broth (LB) to an OD_600_ of ∼0.4 and induced with IPTG to a final concentration of 1 mM and allowed to express Zα for 4 h at 37 °C, then centrifuged to collect the cell pellets. Cell pellets were resuspended in lysis buffer (50 mM Tris-HCl (pH 8.0), 300 mM NaCl, 10 mM imidazole, 1 mM BME) and sonicated. Lysate was centrifuged and the supernatant was applied to a His-trap column, (50 mM Tris-HCl (pH 8.0), 1 M NaCl, 10 mM imidazole, 1 mM BME), and eluted in 20 ml of elution buffer washed with 40 ml of lysis buffer, 80 ml of wash buffer (50 mM Tris-HCl (pH 8.0), 300 mM NaCl, 500 mM imidazole, 1 mM BME). The eluents were concentrated to ∼2 ml and applied to a HiLoad 16/600 Superdex 200 prep grade Gel Filtration Column (GE Healthcare) and the peak corresponding to pure protein (which elutes at ∼80 ml in 20 mM potassium phosphate (pH 6.4), 300 mM NaCl) was collected and concentrated using an Amicon 3 kDa cutoff centrifugal filter (Millipore-Sigma, Burlington, MA). Zα was dialyzed and concentrated into 20 mM potassium phosphate (pH 6.4), 25 mM, 0.5 mM EDTA and concentrated to ∼2 mM using an Amicon 3 kDa cutoff centrifugal filter (Millipore-Sigma). Subsequent dilutions were made in 20 mM potassium phosphate (pH 6.4), 25 mM, 0.5 mM EDTA as needed for the different experiments. We confirmed that all recombinant proteins were free of contaminating nucleic acid (Fig. S5).

### DNA and RNA constructs and preparation

The d(CpG)_3_, d(CpG)_6_, and d(5mCpG)_3_ constructs were synthesized by Integrated DNA Technologies. The r(CpG)_3_ construct was synthesized by Dharmacon (a part of Horizon Discovery). The 8mG4 d(CpG)_3_ construct was synthesized by the Yale School of Medicine oligo synthesis resource. All nucleic acid constructs in this study were heat annealed prior to use at 95 °C for 5 minutes followed by slow cooling at room temperature for 30 min. For circular dichroism (CD) measurements, all of the constructs were 50 μM in 20 mM potassium phosphate, 25 mM NaCl (pH 6.4), 0.5 mM EDTA, or the same buffer but with 6 M NaClO_4_ or a 1:6 M ratio of nucleic acid:Zα.

### Circular dichroism

All CD measurements were collected using a JASCO J-815 CD spectrometer (run using Spectra Manager version 2 (JASCO)) in a 0.1 cm quartz cuvette. Z-form adoption by high-salt and Zα binding was carried out by incubating the constructs in 6M NaClO_4_ or with saturating amounts of Zα at a 1:6 M ratio of nucleic acid:Zα for 1 h at 42 °C before cooling to 25 °C and measuring. Spectra were collected in 1-nm steps from 320 to 220 nm with an average of two scans. All measurements with unmodified nucleic acids as well as d(5mCpG)_3_ and 8mG4 r(CpG)_3_ were conducted twice, and the profiles shown in Figure 3 overlaid with profiles from second independent measurements are provided in Fig. S4. All duplicate profiles confirmed the high reproducibility of the measurements.

## Conclusion

In summary, our study employed a computational combined with an experimental approach that enabled the identification of potential Zα-containing proteins across various organisms. These ZBPs open up new avenues in studies of Zα domains and Z-nucleic acids in virus sensing and innate immunity. The domain architectures within predicted Z-DNA binding proteins imply that they potentially serve functions distinct from those currently attributed to the known ZBPs (Fig. S3). These functions could be conserved in mammals but in the form of synergy between a ZBP and another protein like the cooperative function between ADAR1_p150_ and DHX9 (67). Furthermore, our experimental validation of two Zα-containing proteins confirms not only their presence in currently uncultivated giant viruses but also their functionality. Our findings open new questions regarding the role of Zα domains in these giant viruses and suggest that the interplay between ZBPs/Z-nucleic acid is an evolutionarily ancient feature as shown for the cGAS/STING pathway (68). It is tempting to speculate that the ZBPs we predicted in giant viruses and early diverging metazoan could be involved in an ancient pathogen detection mechanism that could provide functional and evolutionary insight into innate immune signaling pathways in animals. Our validation results underscore the need for heightened caution in Zα domain prediction. We demonstrate that the mere detection of a Zα domain by prediction tools or the observation of structural similarities between predicted wHTH and crystallized Zα domains fall short of substantiating experimental B(A)-to-Z conversion or Z-nucleic acid-binding. These findings emphasize that the ability to bind to Z-nucleic acid should be the definitive criterion for establishing a wHTH as a true Zα domain.

In our study, we focused on validating the Zα and Zα-like domains found mainly in multi-domain proteins. Although we demonstrate that wHTH domains we classify as Zα-like cannot bind Z-nucleic acid nor convert B-to-Z, we cannot exclude the possibility that the proteins with Zα-like domains may still be involved in Z-nucleic biology. This is because other proteins lacking Zα domains have been found to bind Z-DNA (69) or play a role in regulating Z-RNA sensing (70).

## Data availability

The data that support the findings of this study are available from the corresponding authors upon request. The primary sequence and the id-numbers of the new ZBPs and Zα-like containing proteins are summarized in Fig. S3 and Table S6. Other sequences used in this work are available from Frederik Schulz and Mamadou Amadou Diallo upon reasonable request.

## Supporting information

This article contains supporting information.

## Conflicts of interest

The authors declare that they have no conflicts of interest with the contents of this article.

## Figures and Tables

**Supplementary tables caption:**
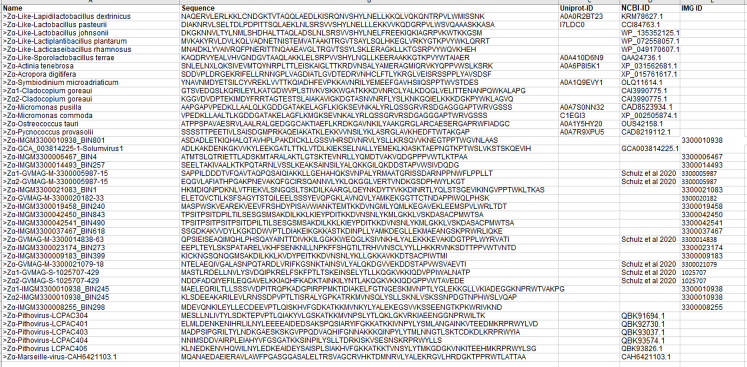


## Supporting information

SI-figureS4_Plots_with_replicates

Supplemental Figure Legends

Revised figures S1–S5
