# Supplementary material for: Novel Z-DNA binding domains in giant viruses
Source: J Biol Chem. 2024 Jun 27;300(8):107504. doi: 10.1016/j.jbc.2024.107504 (PMC11298590; doi:10.1016/j.jbc.2024.107504)
Supplement: SI-figureS4_Plots_with_replicates [file mmc1.pptx]

## Slide 1
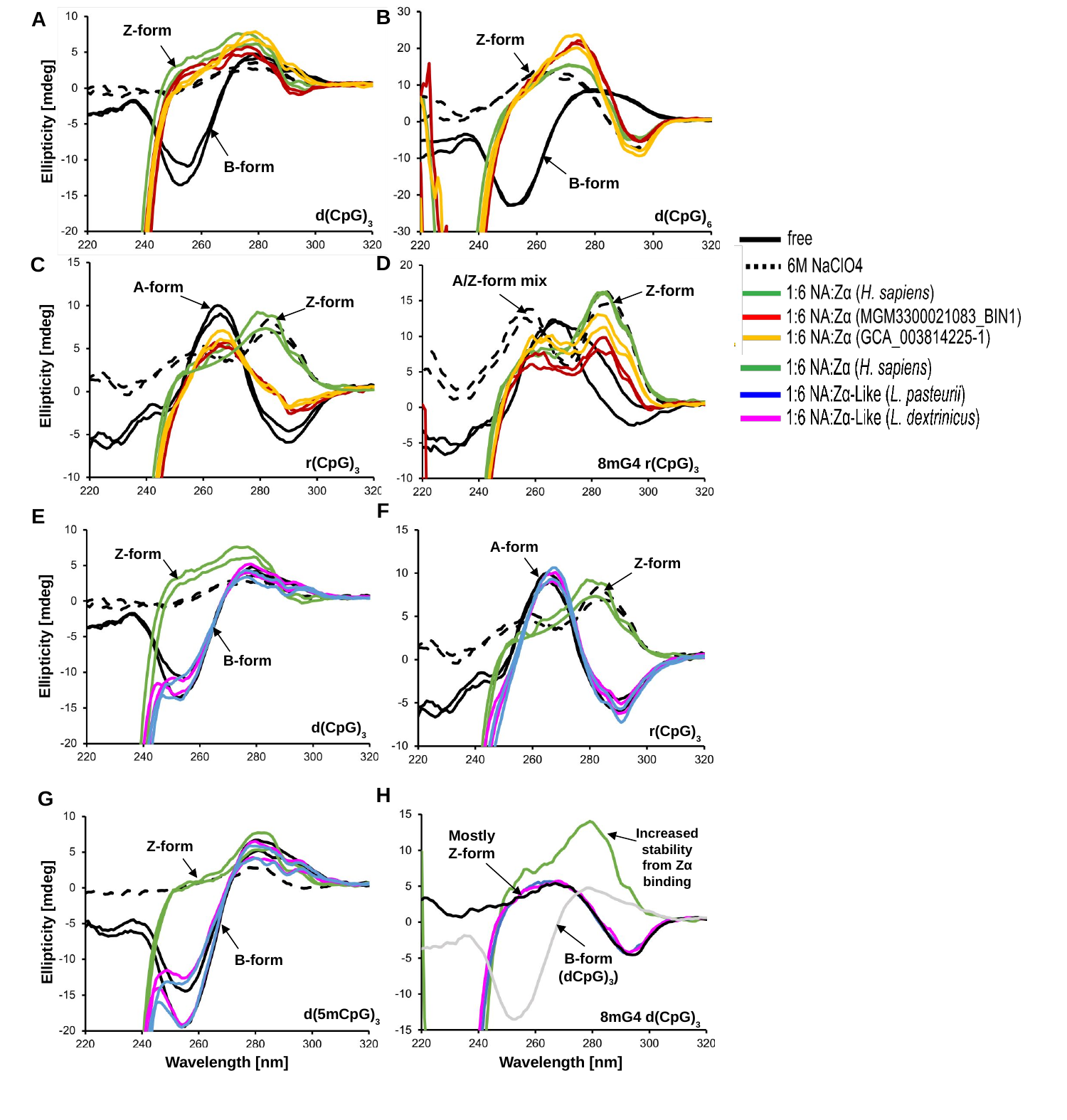

B
A
Z-form
Z-form
Ellipticity [mdeg]
B-form
B-form
d(CpG)3
d(CpG)6
D
C
A/Z-form mix
A-form
Z-form
Z-form
Ellipticity [mdeg]
r(CpG)3
8mG4 r(CpG)3
F
E
A-form
Z-form
Z-form
Ellipticity [mdeg]
B-form
d(CpG)3
r(CpG)3
H
G
Increased stability from Zα binding
Mostly Z-form
Z-form
Ellipticity [mdeg]
B-form (dCpG)3)
B-form
d(5mCpG)3
8mG4 d(CpG)3
Wavelength [nm]
Wavelength [nm]
