# Supplementary material for: Novel Z-DNA binding domains in giant viruses
Source: J Biol Chem. 2024 Jun 27;300(8):107504. doi: 10.1016/j.jbc.2024.107504 (PMC11298590; doi:10.1016/j.jbc.2024.107504)
Supplement: Supplemental Figure Legends [file mmc2.docx]

**Supplemental Figure 1:** Alignment of new Zα with known eukaryotic Zα (HsZα_ADAR1_ and Mm Zα_ADAR1_) and viral Zα (ASFVZα_I73R_ vvZα_E3_ and CyHV-3Zα_ORF112_. The consensus structure Zα domain is shown at the top. The key DNA-interacting residues in ADAR1 (corresponding to Asn174, Tyr177, P192, P193 and Trp195 in HsZα_ADAR1_) are pointed with red arrows. Hydrophobic residues in the hydrophobic core are highlighted in red front. Validated Zα in this study are indicated in green front and those that are validated as Zα-like are in red front.

**Supplemental Figure 2**: Giant viruses' environnemental distribution. Each nucleocytoplasmic large DNA virus (NCLDV) is facing its sampling environment.

**Supplemental Figure 3:** Domains architecture of several representative cases of news ZBPs.

Domains are predicted by SMART^®^.

**Supplemental Figure 4:** Overlay of CD profiles of two independent experiments.

**Supplemental Figure 5:** Purity of expressed Zα domains. (**A**): Purified recombinant proteins were tested for protein contamination by SDS-PAGE using Novex^TM^ 4-20% Tris-Glycine Plus WedgeWell^TM^ Gels. Each lane shows one band corresponding to the correct expected molecular weights shown in the table below. (**B**): Purified recombinant proteins were tested for RNA contamination by spectral analysis using a NanoDrop 2000. Each recombinant protein was free of contaminating nucleic acid as shown by the 260/280 ratios (~0.60 is expected for pure proteins whereas values of ~2.00 is expected for RNA). Full spectral traces for each measurement are provided.

**Supplemental table S6:** Summary of information on the available sequences of Zα and Zα-like used in this study
