# Supplementary material for: Novel Z-DNA binding domains in giant viruses
Source: J Biol Chem. 2024 Jun 27;300(8):107504. doi: 10.1016/j.jbc.2024.107504 (PMC11298590; doi:10.1016/j.jbc.2024.107504)
Supplement: Revised figures S1–S5 [file mmc3.pptx]

## Slide 1
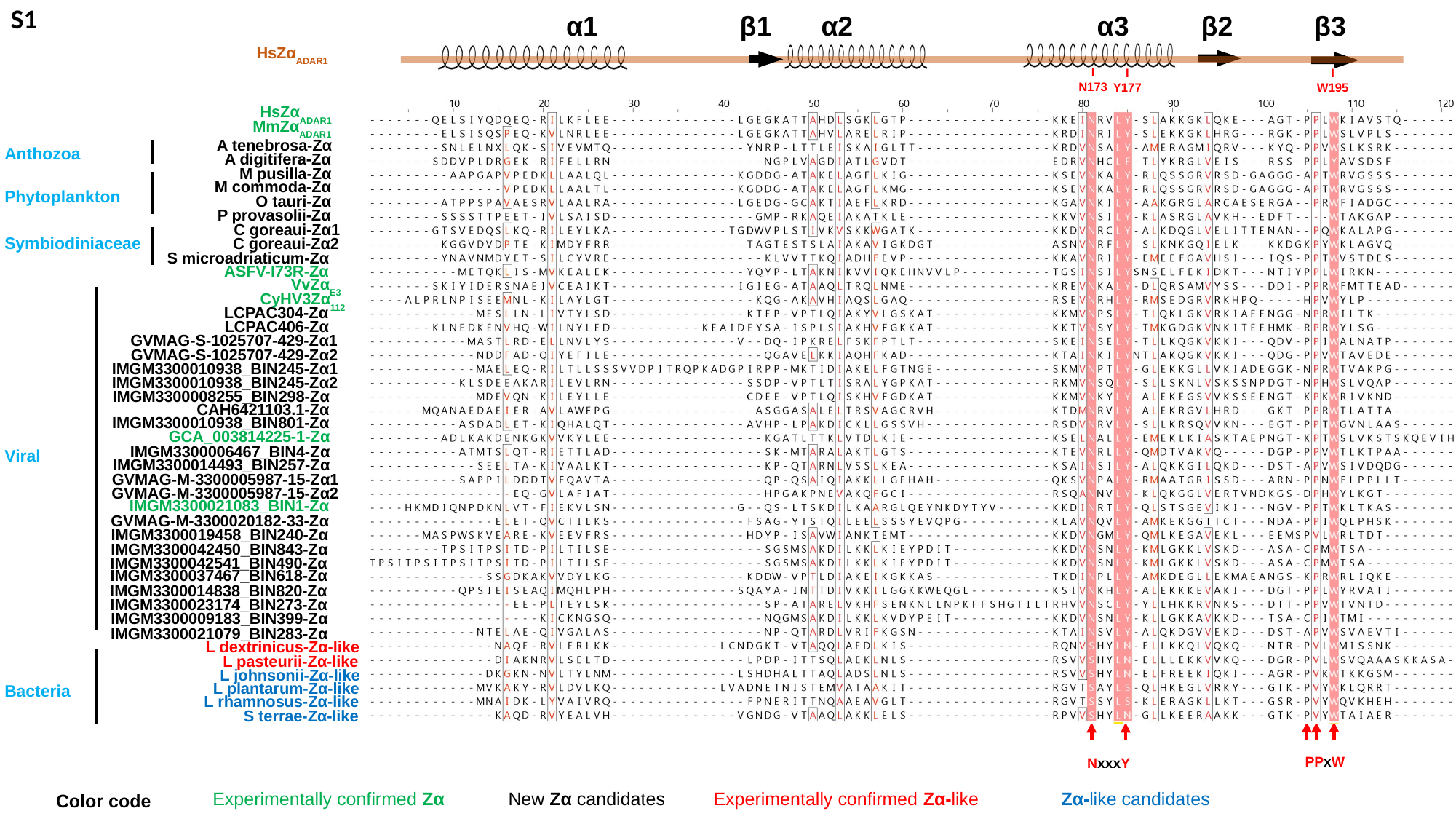

S1
α1
β1
α2
α3
β2
β3
I
N173
I
W195
I
Y177
HsZαADAR1
HsZαADAR1
MmZαADAR1
A tenebrosa-Zα
A digitifera-Zα
M pusilla-Zα
M commoda-Zα
O tauri-Zα
P provasolii-Zα
C goreaui-Zα1
C goreaui-Zα2
S microadriaticum-Zα
ASFV-I73R-Zα
VvZαE3
CyHV3Zα112
LCPAC304-Zα
LCPAC406-Zα
GVMAG-S-1025707-429-Zα1
GVMAG-S-1025707-429-Zα2
IMGM3300010938_BIN245-Zα1
IMGM3300010938_BIN245-Zα2
IMGM3300008255_BIN298-Zα
CAH6421103.1-Zα
IMGM3300010938_BIN801-Zα
GCA_003814225-1-Zα
IMGM3300006467_BIN4-Zα
IMGM3300014493_BIN257-Zα
GVMAG-M-3300005987-15-Zα1
GVMAG-M-3300005987-15-Zα2
IMGM3300021083_BIN1-Zα
GVMAG-M-3300020182-33-Zα
IMGM3300019458_BIN240-Zα
IMGM3300042450_BIN843-Zα
IMGM3300042541_BIN490-Zα
IMGM3300037467_BIN618-Zα
IMGM3300014838_BIN820-Zα
IMGM3300023174_BIN273-Zα
IMGM3300009183_BIN399-Zα
IMGM3300021079_BIN283-Zα
L dextrinicus-Zα-like
L pasteurii-Zα-like
L johnsonii-Zα-like
L plantarum-Zα-like
L rhamnosus-Zα-like
S terrae-Zα-like
Anthozoa
Phytoplankton
Symbiodiniaceae
Viral
Bacteria
PPxW
NxxxY
Experimentally confirmed Zα
New Zα candidates
Experimentally confirmed Zα-like
Zα-like candidates
Color code

## Slide 2
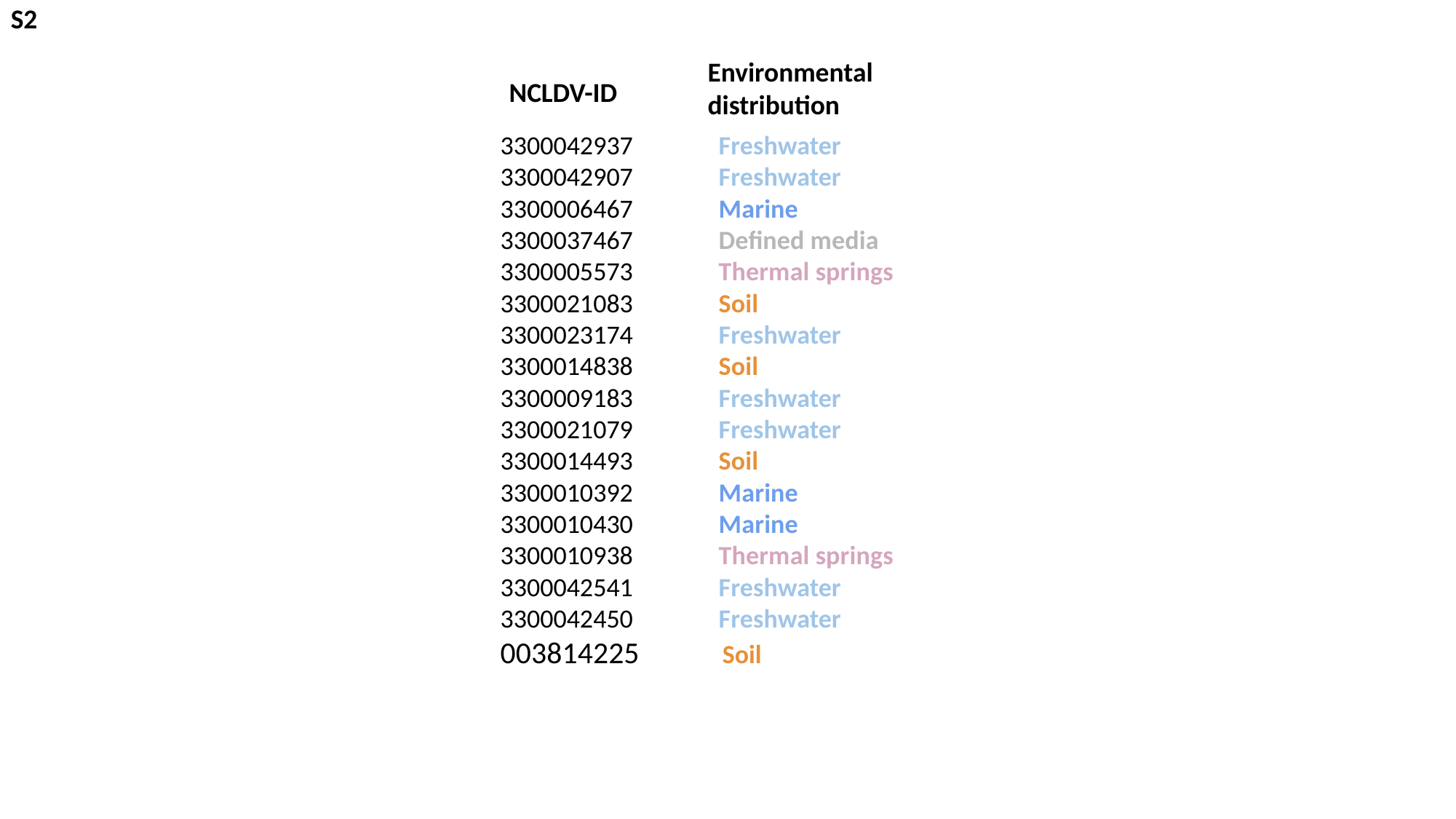

S2
Environmental distribution
NCLDV-ID
3300042937	Freshwater
3300042907	Freshwater
3300006467	Marine
3300037467	Defined media
3300005573	Thermal springs
3300021083	Soil
3300023174	Freshwater
3300014838	Soil
3300009183	Freshwater
3300021079	Freshwater
3300014493	Soil
3300010392	Marine
3300010430	Marine
3300010938	Thermal springs
3300042541	Freshwater
3300042450	Freshwater
003814225 Soil

## Slide 3
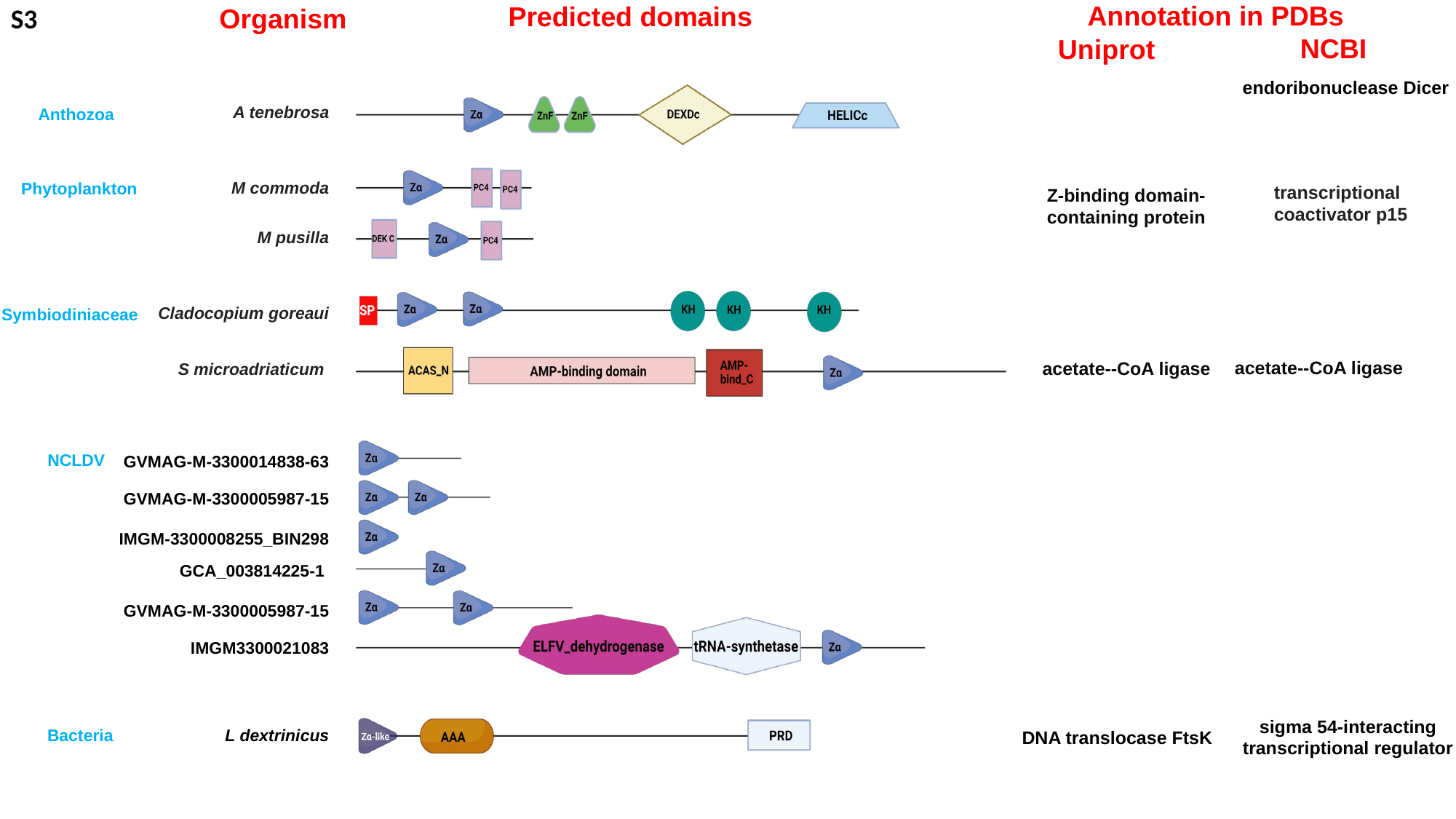

Annotation in PDBs
Predicted domains
Organism
S3
NCBI
Uniprot
endoribonuclease Dicer
A tenebrosa
Anthozoa
M commoda
Phytoplankton
transcriptional coactivator p15
Z-binding domain-containing protein
M pusilla
Cladocopium goreaui
Symbiodiniaceae
acetate--CoA ligase
acetate--CoA ligase
S microadriaticum
NCLDV
GVMAG-M-3300014838-63
GVMAG-M-3300005987-15
IMGM-3300008255_BIN298
GCA_003814225-1
GVMAG-M-3300005987-15
IMGM3300021083
sigma 54-interacting transcriptional regulator
Bacteria
L dextrinicus
DNA translocase FtsK

## Slide 4
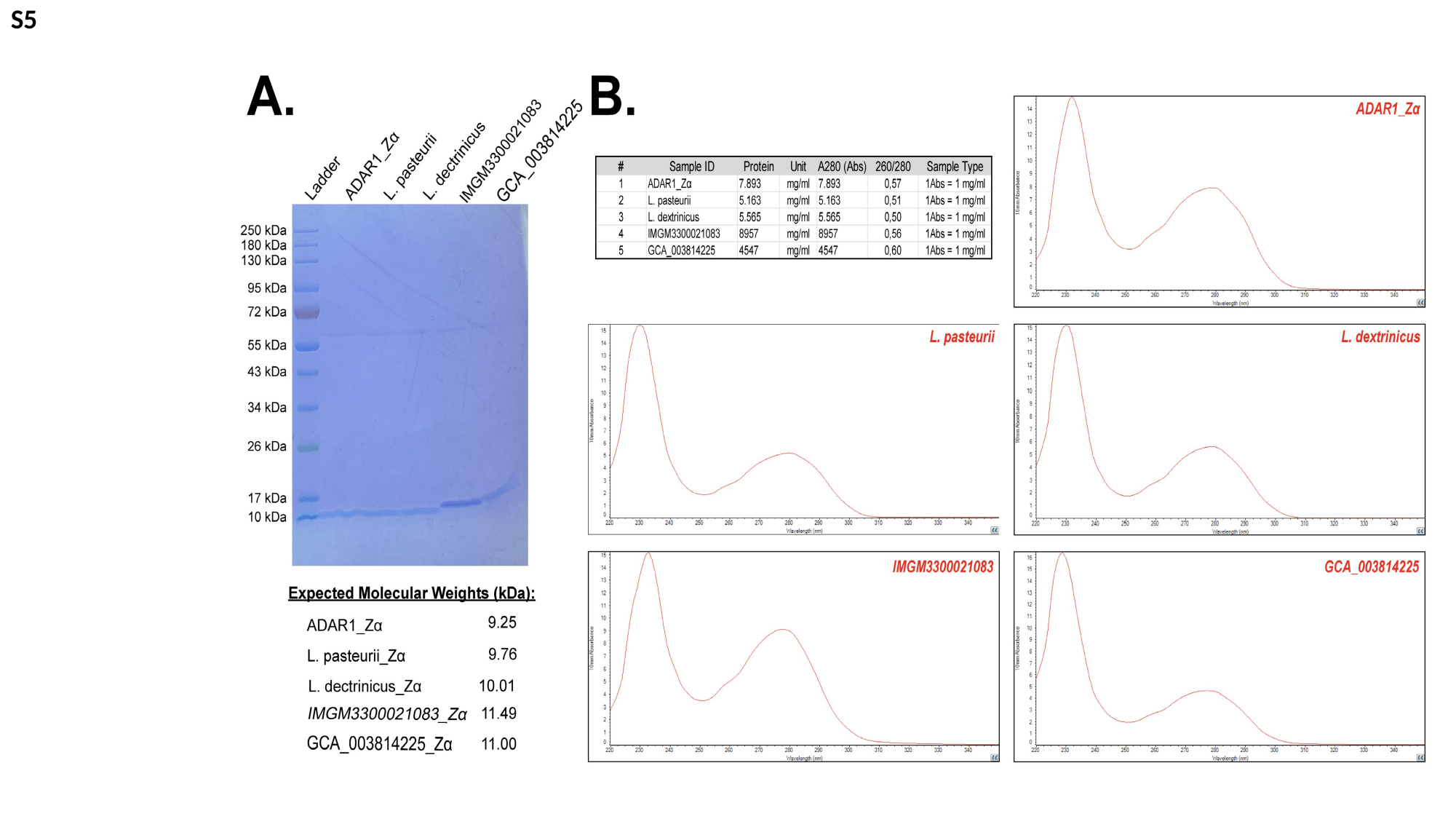

S5
